# Human‐directed attachment behavior in wolves suggests standing ancestral variation for human–dog attachment bonds

**DOI:** 10.1002/ece3.9299

**Published:** 2022-09-20

**Authors:** Christina Hansen Wheat, Linn Larsson, Patricia Berner, Hans Temrin

**Affiliations:** ^1^ Department of Zoology Stockholm University Stockholm Sweden; ^2^ Department of Biology Lund University Lund Sweden

**Keywords:** attachment, dogs, domestication, selection, standing variation, wolves

## Abstract

Domesticated animals are generally assumed to display increased sociability toward humans compared to their wild ancestors. Dogs (*Canis familiaris*) have a remarkable ability to form social relationships with humans, including lasting attachment, a bond based on emotional dependency. Since it has been specifically suggested that the ability to form attachment with humans evolved post‐domestication in dogs, attempts to quantify attachment behavior in wolves (*Canis lupus*) have subsequently been performed. However, while these rare wolf studies do highlight the potential for wolves to express human‐directed attachment, the varied methods used and the contrasting results emphasize the need for further, standardized testing of wolves. Here, we used the standardized Strange Situation Test to investigate attachment behavior expressed in wolves and dogs hand‐raised and socialized under standardized and identical conditions up until the age of testing. We found that 23‐week‐old wolves and dogs equally discriminated between a stranger and a familiar person, and expressed similar attachment behaviors toward a familiar person. Additionally, wolves, but not dogs, expressed significantly elevated stress‐related behavior during the test, but this stress response was buffered by the presence of a familiar person. Together, our results suggest that wolves can show attachment behaviors toward humans comparable to those of dogs. Importantly, our findings demonstrate that the ability to form attachment with humans exists in relatives of the wild ancestor of dogs, thereby refuting claims that this phenotype evolved after dog domestication was initiated.

## Graphical abstract

Disentangling whether specific phenotypes seen in domesticated populations have evolved via novel mutations or are in fact based in standing ancestral variation is of particular importance when we try to understand how domestication might have proceeded and how phenotypes evolve. It has been specifically been suggested that the ability to form attachment (i.e. a social bond based on emotional dependency) with humans evolved post‐domestication in dogs, yet here we show that hand‐raised wolves also express human‐directed attachment. Our findings demonstrate that the ability to form attachment with humans exists in relatives of the wild ancestor of dogs, thereby refuting claims that this phenotype evolved after dog domestication was initiated.

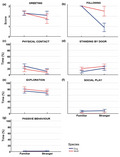

## 1 INTRODUCTION

Domestication, the evolutionary process in which species are selected to live in human‐controlled environments (Price, 2002), has significantly influenced the evolution of behavioral expression in animals (Belyaev et al., 1985; Himmler et al., 2013; Künzl & Sachser, 1999; Trut, 1999). Perhaps the most dramatic example of behavioral evolution during domestication, which has intrigued researchers for centuries, is that of the dog (*Canis familiaris*). The dog was domesticated from now extinct wolf lineages (Bergström et al., 2020; Freedman et al., 2014) 40,000–15,000 years ago, making it the first domestic species (Perri et al., 2021). Because present‐day gray wolves (*Canis lupus*) are widely accepted as an excellent proxy for the common ancestor of dogs (Bergström et al., 2020; Freedman et al., 2014), comparisons between dogs and wolves present a unique opportunity for investigating behavioral evolution, and domestication in particular. Here, we focus on testing whether behavioral traits seen in present‐day dogs uniquely evolved as a result of domestication, or existed as standing variation within pre‐domesticated wolf populations. Quantitative assessment of the relative support for these alternative hypotheses has significant ramifications on our understanding of how domestication might have proceeded, yet such investigations are under‐represented within the canid domestication field. Furthermore, while advances have been made in demonstrating how morphological traits in present‐day domesticated strains are the result of standing variation in wild ancestral populations, mainly in plants (Nesbitt & Tanskley, 2002; Studer et al., 2011), this insight is lacking for more complex phenotypes, such as behavior. Thus, resolving whether behavioral evolution during domestication takes place via the use of existing variation or novel mutations could provide important insight into which behavioral phenotypes might have played a crucial role in the early stages of animal domestication and how these phenotypes have evolved.

As for most domesticated animals, the initial domestication of the dog was likely driven by a simultaneous down‐regulation of aggressive and fearful behaviors and upregulation of social and affiliative behaviors (Belyaev et al., 1985; Künzl & Sachser, 1999; Trut, 1999). While increased expression of human‐directed sociability is generally considered to be more pronounced in domesticated animals when compared to their wild ancestral species (Belyaev, 1979; Hare et al., 2012), the dog (*Canis familiaris*) is probably the domesticated animal that has most successfully adapted to life in a human‐controlled world (Wynne, 2021). Feral dogs throughout the world live in close proximity to humans, and for the smaller proportion of dogs that live in human households as pets, the social relationships with their owners are particularly intimate, with the pet dogs participating in various aspects of their owners' everyday life, or even sleeping in their beds (Udell et al., 2010; Wynne, 2021). Several researchers have built upon the uniqueness of this human–dog relationship, suggesting that dogs can form attachment bonds to their human caregivers comparable to that between a child and its parent (Miklósi & Topal, 2013; Topál et al., 1998, 2005), and that this ability evolved post‐domestication as a trait unique to dogs (Topál et al., 2005).

Attachment is a social bond based on emotional dependency formed between two individuals that endures over time (Ainsworth & Bell, 1970). Originally described as the bond between a human infant and its mother, attachment behavior constitutes any type of behavior performed by an emotionally dependent individual to promote and maintain proximity or contact to the individual of attachment (Ainsworth & Bell, 1970; Bowlby, 1958). Ainsworth's Strange Situation Procedure (SSP) is a highly influential method developed to empirically investigate the attachment bond between human infants and their primary caretaker (Ainsworth & Bell, 1970). Based on the assumption that the attachment system is only activated in challenging situations (Prato Previde & Valsecchi, 2014; Rehn et al., 2013), the SSP examines an infant's attachment behavior toward its primary caregiver under standardized test conditions of interchanging low and high emotional stress situations, which includes separation, reunion, and the presence of a stranger (Ainsworth & Bell, 1970). In the SSP, attachment is quantified and based on the infants expression of (i) safe haven effects; which are expressed through proximity maintenance and contact‐seeking behaviors where the infant actively seeks to maintain physical contact with the attachment figure; and (ii) secure base effects, which can be expressed as the infant's increased willingness to engage in exploratory and/or play behavior when the attachment figure is present (Ainsworth & Bell, 1970). Thus, attachment quantification is based on the infant's ability to discriminate between a primary caretaker and a stranger during the SSP test conditions (Ainsworth & Bell, 1970; Rehn et al., 2013; Topál et al., 1998).

Adaptation of the SSP to dogs (i.e., the Strange Situation Test [SST]) was first performed in a study just over 20 years ago (Topál et al., 1998), wherein the authors, based on the attachment behaviors shown in the SSP, suggested that the human–dog bond is comparable to that of a parent–child attachment bond. Since then multiple studies (Gácsi et al., 2001; Mariti et al., 2013; Solomon et al., 2019; Valsecchi et al., 2010) have used the SST to confirm that dogs express more affiliative behaviors toward their human caregiver, engage in more explorative behaviors in the presence of their human caregiver and express distress behavior upon separation from their human caregiver when compared to a stranger. In 2005, Topál et al. were also the first to compare attachment in dogs and hand‐raised wolves using the SST, finding that wolves did not discriminate between a familiar person and a stranger at 16 weeks of age. The authors suggested that an absence of a direct functional relationship between puppy–mother attachment in wild wolves could explain the wolves' inability to form attachment bonds to their human caregivers and concluded that dogs have evolved the ability to show attachment toward humans post‐domestication. This hypothesis brought forward by Topál et al. (2005) has later been coined the “Attachment Hypothesis” (Range & Marshall‐Pescini, 2022) and represents one hypothesis among a range of dog domestication hypotheses (see Range & Marshall‐Pescini, 2022 for a coherent, updated list). Another dog domestication hypothesis specifically incorporating human–dog attachment is the “Evolutionary Social Competence Hypothesis” (Miklósi & Topal, 2013). This hypothesis is based on the assumption that because dogs are continuously exposed to a prosocial environment with humans, they themselves have evolved unique social human‐like competence through natural and artificial selection during the domestication process. Essential for the “Evolutionary Social Competence Hypothesis” is that the ability to form an attachment with humans is thought to form the very foundation for dogs' special social competence skills (Miklósi & Topal, 2013). Importantly, the common denominator for the “Attachment Hypothesis” and the “Evolutionary Social Competence Hypothesis” is that they both draw heavily upon the assumption that dogs, but not wolves, have a unique ability to form an attachment with humans.

Following the study by Topál et al. (2005), a total of four studies have subsequently investigated human‐directed attachment using hand‐raised wolves (Gácsi et al., 2005; Hall et al., 2015; Lenkei et al., 2020; Ujfalussy et al., 2017). The results from these studies vary considerably in their findings of the expression of attachment in wolves. Gácsi et al. (2005) reported that wolves up to 5 weeks of age expressed aggression and avoidance behavior toward their caregiver; Ujfalussy et al. (2017) reported affiliative behaviors expressed toward a familiar person at 6, 12, and 24 weeks of age with discrimination between familiar and unfamiliar people; and Lenkei et al. (2020) reported that adult wolves expressed increased contact‐seeking behavior and secure base effects in the presence of a familiar person. The remaining study of the four, by Hall et al. (2015), was the only one to use the SST to quantify attachment in wolves and showed that hand‐raised wolf puppies up to the age of 8 weeks expressed attachment in the form of proximity and contact‐seeking toward a human caregiver.

While the majority of these wolf studies indeed highlight the potential for wolves to express attachment behavior toward humans, the varied, and in some cases, contrasting, results also illustrate the need for further testing of wolves in two important ways. First, several different tests and metrics have been used to quantify attachment or social affiliation with humans across these studies, making study comparisons difficult. Second, the contrasting results highlight the importance of hand‐raising and socializing wolves and dogs under identical conditions in order to decrease the risk that even subtle environmental biases affect experimental outcomes. Specifically, socialization procedures vary across wolf studies, with some wolves raised as singles individuals (Gácsi et al., 2005; Lenkei et al., 2020; Miklósi et al., 2003; Topál et al., 2005) and some with their litter mates (Hall et al., 2015; Range et al., 2015; Udell et al., 2008). Therefore, more efforts standardizing and replicating studies on wolves and dogs are essential in furthering our understanding of the behavioral consequences of domestication.

Here, we aim to further contribute to the understanding of how behavioral traits evolve. We will test whether a specific behavioral phenotype, i.e., attachment, is present in both the proxy ancestral species and its domesticated derivate to directly address the question of whether this behavioral trait has evolved via novel mutation on the dog domestication lineage, or if it is present already in the proxy ancestral population. We are thereby directly testing the “Attachment Hypothesis” (Topál et al., 2005) and indirectly the “Evolutionary Social Competence Hypothesis” (i.e., the ability to form attachment is the foundation for dogs' unique human‐like social competence (Miklósi & Topal, 2013)), which both assume that the ability to form an attachment with humans is unique to dogs. We will do so by subjecting 23‐week‐old wolves and dogs that were hand‐raised and socialized under identical, standardized conditions (Klinghammer & Goodman, 1987; Range & Virányi, 2011; Udell et al., 2008) to the SST adapted to canids (Topál et al., 1998, 2005). Specifically, we will quantify safe haven and secure base effects as was done by Topál et al. (1998, 2005) to quantify the expression of attachment behavior in wolves and dogs.

While adult wolves (Lenkei et al., 2020) have been shown to express attachment behavior toward human caregivers in a non‐standardized test, attachment behavior has never been quantified in wolves older than 16 weeks using the standardized SST (Topál et al., 2005). Thus, by testing wolves as old as 23 weeks in the SST, we are here further addressing an age‐related aspect of attachment behavior in wolves. Finally, we will also include a separate, simultaneous quantification of basic stress and fear‐related behaviors throughout the SST to gain novel insight into how wolves and dogs are affected by the test situation.

## 2 MATERIALS AND METHODS

### 2.1 Study site

This study was conducted between the years of 2014 and 2016 at Tovetorp Zoological Fieldstation, Stockholm University, Sweden.

### 2.2 Study animals

Twelve Alaskan huskies and 10 European gray wolves were included in this study. The Alaskan husky is a type of dog specifically bred for dog sledding. While the Alaskan husky is not a registered dog breed, this dog type represents a genetically distinct population of dogs despite its unregulated breeding program, based on a mix of registered dog breeds, which predominantly includes Siberian Husky and Alaskan Malamute and to a lesser extent Saluki and range of pointer breeds (Huson et al., 2010; Thorsrud & Huson, 2021).

The dogs, four females and eight males, came from two unrelated litters of each six puppies. The wolves, five females and five males, came from three different litters, of which two litters were full siblings (six puppies in total) and the third (four puppies) was unrelated to the first two litters. To minimize environmental bias, including maternal effects, which are well documented to affect the development of behavioral patterns (Bray et al., 2017; Clark & Galef Jr, 1982; Wilsson & Sundgren, 1998), puppies were hand raised and extensively socialized under standardized conditions (Table 1) from the age of 10 days by a team of trained caregivers. Caregivers were both male and female and while the number of caregivers totaled nine over the course of the project, the team each year only consisted of four to five, of which four caregivers were the same across years. Dog and wolf litter were raised in separate rooms and enclosures, and socialized with 24‐h presence of caregivers for the first 2 months. Caregiver presence was decreased by a few hours a day from when the puppies were 2 months. A subsequent gradual decrease in caregiver presence followed and at 4 months of age the puppies would spend every other night without a caregiver present. Puppies were reared in identical indoor rooms until they were 5 weeks old and thereafter given access to smaller roofed outdoor enclosures. At 6 weeks of age, after a week of habituation to the roofed outdoor enclosure, puppies were given access to a larger fenced grass enclosure. From the age of 6 weeks, the puppies had free access to all three enclosures during the day and access to the indoor room and the roofed enclosure during the night. At 3 months of age, puppies were moved to large outdoor enclosures (two enclosures of 2000 square meters each for the wolves and dogs), in which they remained for the rest of the study period. Dogs and wolves were kept separate throughout the entire period. Behavioral observations were initiated immediately at 10 days of age and behavioral testing was initiated at 6 weeks of age (i.e., novel object tests, Hansen Wheat et al., 2019). Caregiving, handling and socialization procedures, enrichment, testing procedures, and exposure to the new environments were standardized across all 3 years. Puppies were never disciplined or trained. From the age of 8 weeks, puppies were gradually introduced to strangers through the fence (note that they never met the stranger used in the SST test in this study prior to testing), always with the support of one or more caregivers, and were at the time of the SST accustomed to groups of up to 10–15 strangers.

**TABLE 1:** Study animal protocol. Overview of environment, caregiver presence, behavioral observations, testing, and exposure to strangers experienced at which ontogenetic stages by wolves and dogs in the study.

Condition	10 days to 5 weeks	5–6 weeks	6–12 weeks	12–26 weeks,
Environment	Indoor room	+ roofed, outdoor enclosure	+ grass enclosure	2000 sqm. enclosure
Caregiver presence	24 h	24 h	24 h, gradual decrease from 8 weeks	Continued gradual decrease
Behavioral observations	Yes	Yes	Yes	Yes
Testing	No	No	Weekly, starting at 6 weeks	Weekly, SST at 23 weeks
Exposure to strangers	No	No	Starting at 8 weeks	Yes

### 2.3 Experimental design

All wolves and dogs were tested in the SST at the age of 23 weeks (dogs: 23 weeks ±0 and wolves: 23 weeks ±0.3). The experimental design was identical to that of Topál et al. (2005). Briefly, the SST adapted to dogs consists of seven experimental episodes, each lasting 2 min, in which the presence and absence of a familiar person and a stranger in a test room with the focal animal are alternated (Table 2).

**TABLE 2:** Strange Situation Test procedure. In the seven episodes of the Strange Situation Test, a familiar person (F) and/or a stranger (S) is present in the test room with the focal animal (except for episode 5 where the animal is alone). Each episode is structured differently. The procedure is identical to the study of Topál et al. (2005).

Episode	Present	Minutes	Structure of episode
1	F	0–2	F leads the animal into the test room, closes the door, sits down in one of two chairs, and reads from a paper in silence. After 1 min F initiates play with the animal. F stops playing after 2 min as S enters the room
2	F + S	2–4	S enters the room and stops for 5 s, allowing the animal to greet, and then sits down in the vacant chair. After 30 s S initiates a friendly chat with F. After 30 s S stops chatting with F stands up and initiates play with the animal. F then leaves the room as quietly as possible
3	S	4–6	S continues to play/initiate play with the animal. After 1 min S stops playing and returns to the chair. If the animal initiates contact S is allowed to reciprocate physical contact by petting it
4	F	6–8	F calls the animal from outside the room. After entering the room F stops for up to 5 s to allow the animal to greet and then goes to the chair and sits down. S leaves the room. F initiates play with the animal for 1 min and then returns to the chair. If the animal initiates contact F is allowed to reciprocate physical contact by petting it. At the end of the episode F says “I must go, stay here” and leaves the room
5	–	8–10	The animal is alone in the room
6	S	10–12	S enters the room, stops for up to 5 s to allow the animal to greet, and then initiates play with the animal. After 1 min S sits down in the chair. If the animal initiates contact S is allowed to reciprocate physical contact by petting it
7	F	12–14	F calls the animal from outside the room. After entering the room F stops for up to 5 s to allow the animal to greet. S leaves the room while F invites the animal to play for 1 min and then sits down in the chair. If the animal initiates contact F is allowed to reciprocate physical contact by petting it

The familiar person was a primary, female caregiver, who had raised all the litters from 10 days of age and was the caregiver who had spent the most time with the animals. The female stranger had never met the dogs or wolves prior to the experiment. The same familiar person and stranger were used in all tests. In the 6 × 6 meter test room, which was familiar to both dogs and wolves, two chairs were placed in the middle of the room, 2 m from each other, and facing the same direction. Seven toys from the animal's home enclosure, such as balls, rope, and rubber toys, were distributed across the floor in the test room. Familiar toys were used to avoid the risk of eliciting a neophobic response. Tests were filmed with two diagonally mounted GoPro cameras (model Hero, 3, 3+, 4, GoPro Inc.).

### 2.4 Behavioral scoring—SST

Following the procedures in Topál et al. (2005), a total of seven behaviors were quantified using an ethogram (Table 3). These seven behaviors included: (1) greeting, following, physical contact, and standing by the door—all categorized as *safe haven effects*, which are expressed as a means to maintain proximity or physical contact with the attachment figure (Ainsworth & Bell, 1970); (2) exploration and play—both categorized as *secure base effects*, which can be expressed more in the presence of the attachment figure (Ainsworth & Bell, 1970); and (3) passive behavior—categorized as *other behavior* related to other aspects of the social and physical environment (Topál et al., 2005). Behaviors were further divided into (a) continuously measured behaviors, which included exploration, passive behavior, physical contact, social play, and standing by the door, and (b) scored behaviors, which included the following and greeting (Table 3, Table S1–S10).

**TABLE 3:** Ethogram, SST. Behavioral categories coded following Topál et al. (2005), including (a) continuously measured behaviors and (b) scored behaviors. All continuous behaviors were scored as both frequency and duration.

Behavior	Definition
(a) Continuously measured behaviors	
Exploration	Activity directed toward non‐movable aspects of the test room (not including toys), including sniffing, distal visual inspection (starring or scanning), close visual inspection, or oral examination, while F and/or S are present and during episode 5 when the animal is alone
Passive behavior	Sitting, standing, or lying down without any orientation toward the environment while F and/or S are present, and during episode 5 when the animal is alone
Physical contact	Bodily contact initiated by F or S (e.g., petting and touching) or the animal
Social play	Motor activity performed when interacting with F or S; including running, jumping, active physical contact, and chasing toys
Stand by the door	Standing within 1 m of the door and facing toward the door
(b) Scored behaviors	
Following	Conditional scoring between 0 and 3 of following F and S leaving the room while the other person stays behind. **Score 0:** no orientation toward the leaving person at all, or only for <1 s. **Score 1:** orientation toward the leaving person for >1 s. **Score 2:** following the leaving person to the door. **Score 3:** trying to get through the door or standing by the door for >1 s. The mean based on scores from episodes 3, 4, and 7 is used as the total score
Greeting	The behavior of the animal toward the entering F or S, scored using one of five categories: **approach initiation (+1):** the animal moves toward the entering person; **full approach (+1):** the animal approaches the entering person until physical contact is made; **avoidance (−1):** avoidance behavior toward the entering person, such as backing or getting out of the way of the entering person; **durable physical contact upon greeting (+1/2):** the animal spends >3 s in bodily contact with the entering person; **delay of approach (−1/2):** when F or S enters, the animal hesitates to initiate any form of approach for >5 s. Scores are summed up to a total score (maximum 5 since each person entered twice)

*Note*: F = Familiar person, S = Stranger.

### 2.5 Behavioral scoring—Stress‐ and fear‐related behaviors

As a compliment to the SST, we also scored various behaviors previously used to quantify stress‐ and fear‐related behaviors in wolves and dogs (Cimarelli et al., 2021; Ujfalussy et al., 2017) throughout the test using a separate ethogram (Table 4). Originally, behaviors such as crouching, growling, piloerection, pacing, retreating, startle, tail tuck, and yawning were included in this ethogram. However, with the exception of pacing, crouching, and tail tuck, all other behaviors occurred so rarely, or not at all, that we chose to exclude these behaviors from further consideration (but see Table S2 for full quantification of stress‐ and fear‐related behaviors). Pacing, crouching, and tail tuck were all quantified as state behaviors using both duration and frequency for extraction of fractions and was not necessarily mutually exclusive.

**TABLE 4:** Ethogram, stress, and fear behaviors. Behavioral categories coded for stress and fear behaviors occurring as states and events during the SST.

Behavior	Definition
Crouching	Lowered body position in which the back is curved. Can be accompanied by tucking of the tail
Pacing	Walking or trotting at a steady speed without any exploratory purpose or obvious focus on the surroundings
Tail tuck	The tail is tucked down between the hind legs, and the tail might touch the underside of the stomach

Behavioral scoring for both attachment and stress‐ and fear‐related behaviors was carried out using the software program BORIS v.2.97 (Friard & Gamba, 2016). For the SST of the recorded tests, 25% were independently coded by two of the authors. Cronbach's alpha was calculated and inter‐observer reliability was high for all continuous behaviors (exploration: 0.986; passive behavior: 0.978; social play: 0.985; stand by the door: 0.989; and physical contact: 0.987).

### 2.6 Statistical analyses

For all statistical analyses, we used the software SPSS Statistics v.25.

To account for slight variations in durations of episodes across tests (because of the time it took for the test persons to enter and exit the room), we used the relative proportion of the time spent on each behavior for every episode for all individuals. Upon testing for normality using a Shapiro–Wilk test (Razali & Wah, 2011), we found that the two variables, passive behavior and social play, were not normally distributed. We therefore arcsine transformed these two variables prior to statistical testing. Arcsine transformation is commonly used for proportional data (Cohen et al., 1983; McKillup, 2012). For one wolf the test was aborted prematurely and as a result, episodes 6 and 7 were excluded for this individual, i.e., the sample size is *N* = 9 for the wolves in some comparisons (Table S1). We present the mean and SE for the untransformed data in our figures (McDonald, 2009, Table S2).

The behaviors like greeting, following, physical contact, standing by the door, exploration, social play, and passive behavior were divided into two main categories: (1) “In the presence of the familiar person,” which refers to those episodes in which the familiar person was present (1, 2, 4, and 7); and (2) “In the presence of the stranger”, which refers to those episodes where the stranger was present (2, 3, 6). For the three behaviors, pacing, crouching, and tail tuck, the results are given for each of the seven episodes. We used a general linear model (GLM) for repeated measurements where the proportions of the presence of the familiar person and the stranger were classified as the within‐subject factor, and dogs and wolves as the between‐subject factor. The least significant difference (LSD) pairwise multiple‐comparison test, i.e., multiple‐individual t‐tests between all pairs of groups, was used for the results in Table 5.

**TABLE 5:** Post hoc comparisons of episodes for stress and fear behaviors

Episode	Episode comp.	Mean diff.	SE	*p*	95 CI low	95 CI up
(a) Pacing						
**4**	1	−1.015	0.461	.059	−2.078	0.048
	2	−1.204	0.214	***>.001***	−1.696	−0.711
	3	−1.507	0.291	***.001***	−2.179	−0.835
	5	−0.943	0.277	***.009***	−1.583	−0.303
	6	−1.843	0.436	***.003***	−2.848	−0.838
	7	0.868	0.262	***.011***	0.263	1.473
**7**	1	−1.883	0.343	***.001***	−2.675	−1.091
	2	−2.072	0.328	***>.001***	−2.828	−1.315
	3	−2.375	0.209	***>.001***	−2.856	−1.894
	5	−1.811	0.309	***>.001***	−2.523	−1.1
	6	−2.711	0.555	***.001***	−3.991	−1.431
(b) Crouching						
**2**	1	5.860	2.386	**.04**	0.359	11.361
	3	5.173	2.192	**.046**	0.118	10.228
	4	5.860	2.386	**.04**	0.359	11.361
	5	5.860	2.386	**.04**	0.359	11.361
	6	4.013	2.167	.101	−0.984	9.01
	7	5.860	2.386	**.04**	0.359	11.361
**6**	1	1.847	0.801	*.05*	0.001	3.693
	3	1.16	0.635	.105	−0.304	2.625
	4	1.847	0.801	*.05*	0.001	3.693
	5	1.847	0.801	*.05*	0.001	3.693
	7	1.847	0.801	*.05*	0.001	3.693
(c) Tail tuck						
**2**	1	2.206	1.224	.109	−0.617	5.029
	3	1.927	1.291	.174	−1.05	4.904
	4	2.246	1.196	.097	−0.512	5.003
	5	2.303	1.181	.087	−0.421	5.027
	6	0.632	1.457	.676	−2.727	3.992
	7	2.842	1.178	**.042**	0.125	5.559
**6**	1	1.574	0.65	**.042**	0.074	3.074
	3	1.295	0.497	**.031**	0.149	2.441
	4	1.613	0.614	**.03**	0.197	3.03
	5	1.670	0.648	**.033**	0.176	3.164
	7	2.21	1.068	.072	−0.254	4.674

*Note*: The least significant difference (LSD) pairwise multiple‐comparison test for the relevant stress and fear behaviors in wolves. Given for each behavior is episode, episode comparison (comp.), mean difference (diff.), standard error (SE), *p*‐value (*p*), and 95% confidence intervals (low and up). Significant *p*‐values are given in bold italic.

### 2.7 Ethical statement

Daily care and all experiments were performed in accordance with guidelines and regulations under national Swedish Law. The experimental protocols in this study were approved by the Ethical Committee in Uppsala, Sweden (approval number: C72/14). Facilities and daily care routines were approved by the Swedish National Board of Agriculture (approval number: 5.2.18–12,309/13). As required by national law in Sweden, all caregivers working with the puppies were ethically certified and trained to handle animals.

## 3 RESULTS

### 3.1 Attachment behaviors—safe haven effects

#### 3.1.1 Greeting

**FIGURE 1:**
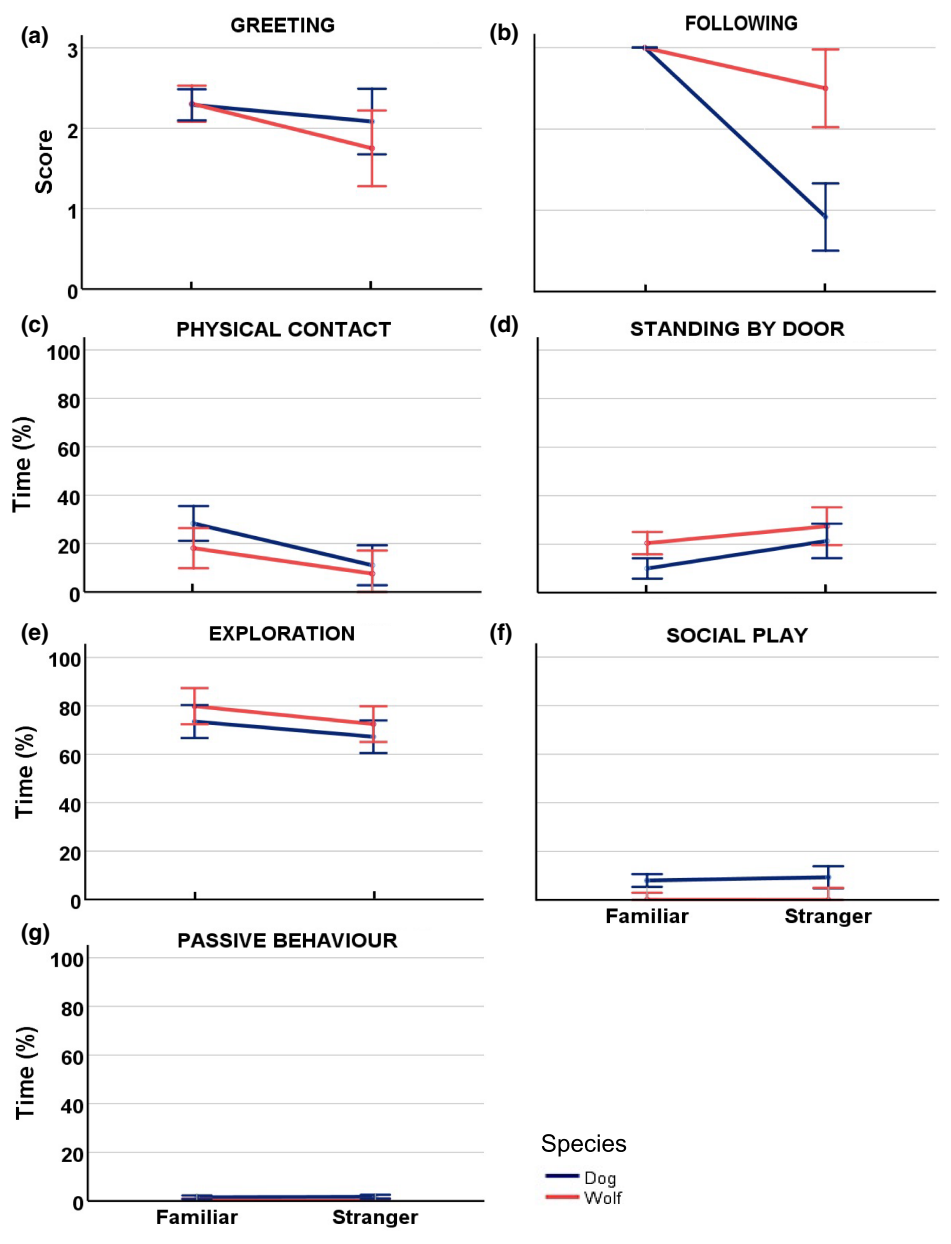
Attachment behaviors, discrimination between familiar person and stranger. Mean score for dogs (blue) and wolves (orange) for (a) greeting and (b) following a familiar person and a stranger, mean proportion of time dogs and wolves spent on (c) physical contact, (d) standing by the door, (e) exploration, (f) social play, and (g) passive behavior, in the presence of a familiar person and a stranger. Error bars represent 95% confidence intervals. See Table S3 for all statistical outputs.

The greeting was only scored when a familiar person or a stranger entered the room, which occurred during episodes 2, 4, 6, and 7. Greeting was expressed significantly more toward the familiar person than toward the stranger (*F*_1,19_ = 10.225, *p* = .005, *N*_wolf_ = 9, *N*_dog_ = 12, Figure 1a, Table S3). There was no difference between dogs and wolves in their expressions of greeting behavior (*F*_1,19_ = 0.637, *p* = .435, Figure 1a, Table S3).

#### 3.1.2 Following

The familiar person was significantly more likely to be followed when leaving the room compared to when the stranger left the room by both dogs and wolves (*F*_1,19_ = 73.134, *p* < .001, Figure 1b, Table S3). There was a species difference in the proportion of following (*F*_1,19_ = 27.473, *p* < .001, Table S3), with wolves showing a higher proportion of following the stranger than dogs. However, both wolves (paired t‐test: *t*_9_ = 2.683, *p* = .028, Table S4) and dogs (*t*_12_ = 9.449, *p* < .001, Table S4) followed the familiar person significantly more than the stranger. There was a significant interaction effect between species and test person (*F*_1,19_ = 27.473, *p* < .001, Table S3), suggesting that the difference between a familiar person and a stranger was greater in dogs than in wolves.

#### 3.1.3 Physical contact

Physical contact with the familiar person was significantly more common than with the stranger (*F*_1,20_ = 12.223, *p* = .002, *N*_wolf_ = 9, *N*_dog_ = 12, Figure 1c, Table S3). Wolves and dogs did not differ in their expression of physical contact (*F*_1,20_ = 2.914, *p* = .104, Table S3).

#### 3.1.4 Standing by the door

Both wolves and dogs stood more by the door when the stranger was in the room and the familiar person was absent, than when the familiar person was in the room (*F*_1,20_ = 18.346, *p* < .001, Figure 1d, Table S3). Wolves stood more by the door compared to dogs (*F*_1,20_ = 5.391, *p* = .031, Table S3), but there was no interaction (*F*_1,20_ = 1.050, *p* = .318, Table S3).

### 3.2 Attachment behaviors—secure base effects

#### 3.2.1 Exploration

Exploration was significantly more common in the presence of the familiar person than in the presence of the stranger (*F*_1,20_ = 7.968, *p* = .011, *N*_wolf_ = 10, *N*_dog_ = 12, Figure 1e, Table S3). There was no difference between wolves and dogs in the expression of explorative behavior (*F*_1,20_ = 1.928, *p* = 0.180, Table S3).

#### 3.2.2 Social play

The expression of social play was not affected differently by the presence of the familiar person and the stranger (*F*_1,20_ = 0.371, *p* = 0.549, *N*_wolf_ = 10, *N*_dog_ = 12, Figure 1f, Table S3). Dogs were significantly more playful than wolves (*F*_1,20_ = 12.761, *p* = .002, Table S3), but there was no interaction (F_1,20_ = 0.4443, *p* = .513, Table S3).

### 3.3 Other behaviors

#### 3.3.1 Passive behavior

The expression of passive behavior was low in both wolves and dogs and not affected differently by the presence of the familiar person and the stranger in either species (*F*_1,20_ = 0.053, *p* = .821, *N*_wolf_ = 10, *N*_dog_ = 12, Figure 1g, Table S3). Dogs were significantly more passive than wolves (*F*_1,20_ = 14.396, *p* = .001, Table S3), but there was no interaction (*F*_1,20_ = 0.112, *p* = 0.741, Table S3).

### 3.4 Stress‐related behaviors

#### 3.4.1 Pacing

**FIGURE 2:**
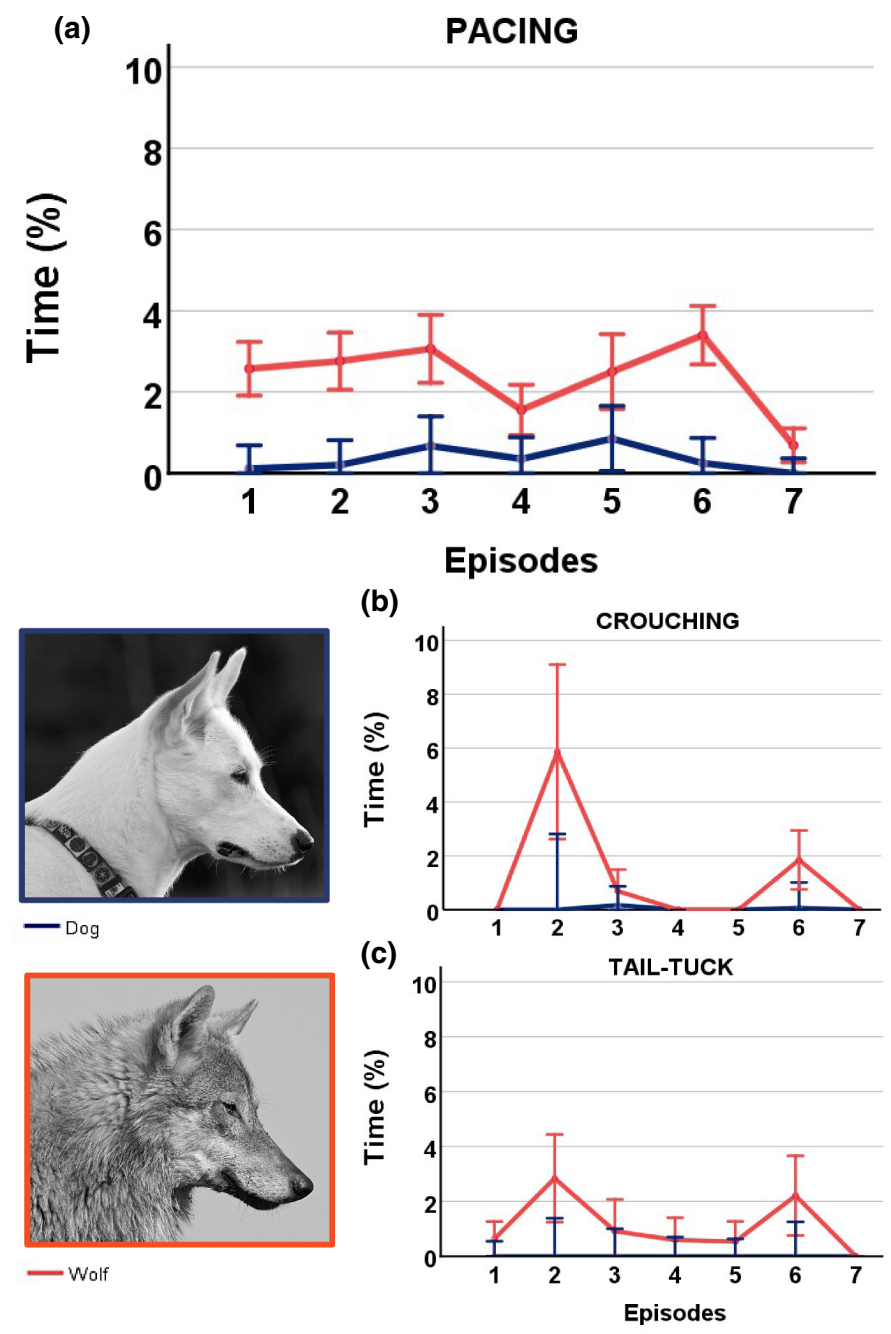
Stress and fear behaviors during the SST. Mean proportion of (a) pacing, (b) crouching, and (c) tail tucking occurring in each of the seven episodes in the SST in dogs (blue) and wolves (orange). Error bars represent 95% confidence intervals. See Tables S5–S10 for all statistical outputs. Photo credit: Christina Hansen Wheat.

There was a significant difference in the expression of pacing between the seven episodes (*F* = 8.528, *p* < .001, df = 6, Figure 2a, Table S5), and there was a significant difference between dogs and wolves (*F* = 48.101, *p* < .001, df = 1, Table S5). There was no difference in pacing between the seven episodes in dogs (*F* = 1.645, *p* = .149, df = 6, Table S6). In wolves, there was an overall difference in pacing among the seven episodes (*F* = 10.449, *p* < .001, df = 6, Table S7). Compared to all other episodes, wolves were pacing significantly less in episodes 4 and 7 when they were reunited with the familiar person after having been alone with the stranger (Figure 2a, Table 5a, see Table S7 for all post hoc comparisons). The only exception was episode 1, in which the wolves had just entered the test room alone with the familiar person, and episode 4, the first reuinion episode. Here, the difference in pacing was not significant (*p* = .059, Figure 2a, Tables 5, S7).

### 3.5 Fear‐related behaviors

#### 3.5.1 Crouching

There was a significant difference in the expression of crouching between the seven episodes (*F* = 7.256, *p* < .001, df = 6, Figure 2b, Table S5) and there was a difference between dogs and wolves (*F* = 9.005, *p* = .007, df = 1, Table S5). There was no difference in crouching between the seven episodes in dogs (*F* = 0.882, *p* = .513, df = 6, Table S8). In wolves, there was an overall difference in crouching between the seven episodes (*F* = 5.414, *p* < .001, df = 6, Table S9). Crouching behavior was only expressed in wolves in episodes in which the stranger was present. Compared to all other episodes, wolves were crouching significantly more in episode 2 when the stranger entered the test room for the first time (Figure 2b, Table 5b, see Table S9 for all post hoc comparisons of episodes). The only exception was episode 6, in which a stranger enters the room for the second time (*p* = .101, Table 5b, Table S9). There was also a noticeable peak in crouching behavior in episode 6 compared to all other episodes, except for episode 2, but while the majority of *p*‐values were just at the .05 level and a few above, none were significant (Figure 2b, Tables 4b, S9).

#### 3.5.2 Tail tuck

There was a significant difference in the expression of tail tuck between the seven episodes (*F* = 4.210, *p* = .001, df = 6, Figure 2c, Table S5) and there was a difference between dogs and wolves (*F* = 5.542, *p* = .029, df = 1, Table S5). This behavior was exclusively expressed in wolves and there was an overall difference in tail tuck between the seven episodes (*F* = 3.102, *p* = .012, df = 6, Table S10). There was a peak in the expression of tail tucking in the episodes when the stranger entered the room (episodes 2 and 6). However, tail tucking was significantly more expressed in episode 6 compared to any other episode, except for episodes 2 and 7 (Figure 2c and Table 5c, see Table S10 for all post hoc comparisons of episodes). Tail tucking in episode 2 was significantly higher than in episode 7, which is the episode when the familiar person re‐entered the room (Figure 2c, Tables 5c, S10).

## 4 DISCUSSION

Here we show how a behavioral phenotype, the ability to express attachment behavior toward a human caregiver, is present in a proxy of the ancestral species of the domesticated dog. Specifically, we demonstrate that 23‐week‐old wolves and dogs equally discriminate between a stranger and a familiar person, and show similar attachment behaviors toward a familiar person through the expression of safe haven and secure base effects. Additionally, while wolves, but not dogs, expressed significantly elevated stress‐related behavior during the test in the form of pacing, this stress response was buffered by the presence of a familiar person. Wolves also expressed quantifiable fear responses to stranger, whereas no such response was detectable in dogs. Together, our results suggest that wolves can express attachment behaviors toward humans comparable to that of dogs. Importantly, our findings demonstrate that the ability to express attachment behavior toward humans exists in relatives of the wild ancestor of dogs, thus refuting claims brought forward in the “Attachment Hypothesis” and the “Evolutionary Social Competence Hypothesis” that this behavioral phenotype is unique to post‐domestication dog lineages.

Our results thus represent a stark contrast to an earlier study by Topál et al. (2005), who upon comparing 16‐week‐old wolves and dogs found that wolves did not discriminate in their expressed attachment behavior toward a human caregiver and a stranger while dogs did discriminate in favor of the caregiver. The authors suggested that wolves did not have the need for a strong bond with their mother in the wild after 6–8 weeks of age, and this could explain their lack of attachment to a human caregiver at the age of 16 weeks. Yet, here we demonstrate that wolves aged 23 weeks of age are capable of discriminating between a stranger and a familiar person and expressing attachment behavior toward a human caregiver. Our results are further supported by two previous studies documenting that wolves aged up to 24 months (Ujfalussy et al., 2017) and wolves older than 1.5 years of age (Lenkei et al., 2020) also express attachment behaviors toward their human caregivers in other test set‐ups than the SST. Together, these studies show that increased independence during ontogeny does not affect attachment behaviors expressed toward human caregiver in wolves. Furthermore, it is important to note that the wolves in the study by Topál et al. (2005) had been relocated to an animal park up to 2 months before the SST test was conducted (Hall et al., 2015; Virányi et al., 2008). Thus, at the time of testing, dogs were still living with their caregivers, but wolves were not, i.e., wolves and dogs were not kept under similar conditions prior to testing. Because environmental factors significantly affect behavioral development (Zimen, 1987) and experimental outcomes in studies comparing wolves and dogs (Hare et al., 2002; Udell et al., 2008), this environmental difference between the wolves and dog in the study by Topál et al. (2005) and our study could be one explanation for the contrasting results.

Our results indicate that the attachment system toward the familiar person was activated in our 23‐week‐old wolves during the SST, and the wolves expressed attachment behavior comparable to those reported in adult dogs (Gácsi et al., 2001; Prato‐Previde et al., 2003; Topál et al., 1998), chimpanzees (van IJzendoorn et al., 2009) and human infants (Ainsworth & Bell, 1970) when subjected to the SST or SSP. Specifically, our wolves expressed safe haven and secure base effects similar to that of identically raised dogs toward, or in the presence of, the familiar person, but not the stranger, which included significantly pronounced contact seeking, proximity maintenance, and exploration behaviors. The expression of passive behavior and social play was limited in both wolves and dogs. Although dogs expressed higher levels of both those behaviors compared to wolves, the expression of neither passive behavior nor social play was dependent on the presence of the familiar person or the stranger in either species. We note that the limited occurrence of passive behavior and social play during the test might have impaired our ability to adequately detect a difference in these behavioral expressions in the presence of a familiar person compared to a stranger (Rehn et al., 2013). Additionally, wolves engaging in play with a human are likely rare (Hansen Wheat & Temrin, 2020) and wolf hybrids show significantly decreased expression of human‐directed play behavior when compared with dogs (Hansen Wheat et al., 2018). Therefore, the wolves' limited engagement in social play during the SST is likely based on a general lack of interest in human‐directed play and does not necessarily represent an expression of low attachment (i.e., secure base effect) to the familiar person.

In dogs, acute stress responses to solitary, restricted confinement can manifest as repetitive movement patterns such as pacing (Beerda et al., 1997). While the test situation in the SST is not strictly solitary per se, as humans are present for the majority of the test, the dogs and wolves were separated from their littermates for the purpose of the test and confined to the restricted space of the test room. The wolves had an exaggerated stress response to the test situation compared to the dogs and expressed significantly increased pacing behavior throughout the test. However, in the two reunion episodes in which the familiar person re‐enters the room after the wolves had been alone with the stranger, the pacing was significantly reduced. This notable reduction in stress response suggests that the familiar person acted as a social buffer for the wolves in an aversive situation (Hennessy et al., 2009). The facilitation of comforting effects in stressful situations by familiar conspecifics is well‐known in various species (von Holst, 1998; Hennessy et al., 2006) and has recently been demonstrated among captive wolf pack members (Cimarelli et al., 2021). Social buffering is believed to be related, although not identical, to attachment and secure base effects (Lenkei et al., 2020), and the social buffering demonstrated in our study thereby strengthens our main conclusion that wolves are capable of showing attachment to a human caregiver.

Furthermore, wolves, but not dogs, had a quantifiable fear response to the stranger entering the test room, expressed as pronounced crouching and tail tucking. This is in line with previous findings of strangers, but not familiar humans, eliciting crouching and tail tucking in hand‐raised wolves (Ujfalussy et al., 2017) and further lends support to the general assumption that hand‐raised wolves do not generalize their socialization to strangers (Klinghammer & Goodman, 1987; Zimen, 1987) as dogs do (Udell, 2015). Dogs on the other hand have been found to express increased motivation to seek social interactions with unfamiliar humans compared to wolves (Bentosela et al., 2016), and recent evidence suggests a possible genetic basis for dogs' hypersociality toward humans (vonHoldt et al., 2017). This “Hypersociability Hypothesis” is another dog domestication hypothesis, and it could explain the absence of stress‐ and fear‐related responses in the dogs in the test situation.

While we present results based on a limited number of wolves and dogs, there is no reason why this should affect the implications of our findings. Specifically, because our results provide proof of concept by demonstrating the presence of attachment behaviors toward a human caregiver in wolves, the number of tested wolves is not essential for their interpretation. Importantly, our results for the wolves suggest that the ability to express human‐directed attachment behaviors was present within pre‐domestication wolf populations, and can therefore stand alone.

In sum, our results add to a slowly, but steady, growing collection of evidence that wolves are capable of expressing attachment behavior toward human caregivers (Hall et al., 2015; Lenkei et al., 2020; Ujfalussy et al., 2017). Additionally, this body of work now highlights that wolves across a wide range of ontogenetic stages, and not just young wolf puppies, possess this capability. Together, the collective evidence from these wolf studies strongly suggests that this behavioral phenotype exists as standing variation in ancestral populations of the domestic dog, hence the narrative that the ability to express attachment behavior toward humans is exclusive to dogs is no longer tenable.

## AUTHOR CONTRIBUTIONS

**Christina Hansen Wheat:** Conceptualization (equal); data curation (equal); formal analysis (equal); methodology (lead); project administration (equal); writing – original draft (lead); writing – review and editing (lead). **Linn Larsson:** Data curation (equal); formal analysis (equal); project administration (supporting); writing – review and editing (supporting). **Patricia Berner:** Methodology (supporting); project administration (supporting); writing – review and editing (supporting). **Hans Temrin:** Conceptualization (equal); data curation (supporting); formal analysis (equal); methodology (supporting); project administration (equal); supervision (lead); writing – original draft (supporting); writing – review and editing (equal).

## FUNDING INFORMATION

This research did not receive any specific grants from funding agencies in the public, commercial, or not‐for‐profit sectors.

## CONFLICT OF INTEREST

The authors declare no conflicts of interest.

## Supporting information


Tables S1–S10

## Data Availability

The data that supports the findings of this study are available in the supplementary material of this article
