# Corrigendum

**DOI:** 10.1002/ece3.9412

**Published:** 2022-10-24

In the recent article by Hansen Wheat et al. (2022), the Acknowledgment section has been updated as follows in the online version:
